# The Effects of a Novel Nanohydroxyapatite Gel and Er: YAG Laser Treatment on Dentin Hypersensitivity

**DOI:** 10.3390/ma16196522

**Published:** 2023-09-30

**Authors:** Demet Sahin, Ceren Deger, Burcu Oglakci, Metehan Demirkol, Bedri Onur Kucukyildirim, Mehtikar Gursel, Evrim Eliguzeloglu Dalkilic

**Affiliations:** 1 Department of Periodontology, Faculty of Dentistry, Bezmialem Vakif University, 34093 Istanbul, Turkey; mihtikar@gmail.com; 2 Department of Restorative Dentistry, Faculty of Dentistry, Bezmialem Vakif University, 34093 Istanbul, Turkey or cdeger@bezmialem.edu.tr (C.D.); or boglakci@bezmialem.edu.tr (B.O.); or edalkilic@bezmialem.edu.tr (E.E.D.); 3 Department of Mechanical Engineering, Yildiz Technical University, 34349 Istanbul, Turkey; demirkol@yildiz.edu.tr (M.D.); or kucukyil@yildiz.edu.tr (B.O.K.)

**Keywords:** nanohydroxyapatite, fluoride varnish, laser, dentin hypersensitivity, surface roughness

## Abstract

Purpose: This study evaluates the effects of a novel nanohydroxyapatite gel and Er: YAG laser on the surface roughness, surface morphology, and elemental content after dentin hypersensitivity treatments. Methods: Dentin discs (2 × 3 × 3 mm^3^) were prepared from 75 human molars. Out of 75 human molars, 50 were used to evaluate surface roughness and randomly divided into five groups: Group ID (intact dentin), Group DD (demineralized dentin), Group BF (fluoride varnish/Bifluorid 10), Group Lsr (Er: YAG laser-50 mJ, 0.50 W, 10 Hz), and Group NHA (nanohydroxyapatite-containing gel). Dentin hypersensitivity was stimulated by 35% phosphoric acid for 1 min (except Group ID). The surface roughness (Ra, μm) was measured via contact profilometry (n = 10). Out of the 75 sound human molars, 25 were used to evaluate the surface morphology and elemental content using scanning electron microscopy and energy-dispersive X-ray spectroscopy (n = 5). The data were statistically analyzed using Welsch ANOVA, Games–Howell, Kruskal–Wallis, and Dunn tests (*p* < 0.05). Results: Group Lsr showed significantly lower surface roughness than Group NHA and Group BF (*p* < 0.05). The SEM analysis indicated that most of the dentinal tubules were obliterated for Group NHA. Precipitant plugs with partially occluded dentinal tubules were observed for Group BF, while partially or completely occluded tubules with a melting appearance were detected for Group Lsr. The EDS analysis revealed that Group NHA and Group Lsr presented similar calcium and phosphorus amounts to Group ID. All dentin hypersensitivity treatment methods could provide promising results in terms of tubular occlusion efficiency. However, laser treatment resulted in smoother surfaces, which could help prevent dental plaque accumulation.

## 1. Introduction

Dentin hypersensitivity is a frequent clinical condition, and it has multifactorial etiology [1]. Dentin hypersensitivity is the pain that derives from exposed dentin in response to chemical, thermal tactile, or osmotic stimuli which cannot be explained by other dental problems [2]. If a patient experiences dentin hypersensitivity, brushing the affected teeth or consuming cold food and beverages could cause pain, and the individual may therefore avoid those practices [3]. Dentin hypersensitivity prevalence is reported to affect between 4% and 69% of the adult population [4], with an average prevalence of 57%. It mostly affects individuals aged between 20 and 40 years [5] and more commonly afflicts women [6]. Dentin hypersensitivity is more prevalent among periodontally compromised patients (60–98%). Buccal surfaces clearly show the highest prevalence rates [6]. The number, size, and diameter of the patent dentinal tubules determine the level of sensitivity that individuals experience [7]. The activated nerve endings generate a severe, sharp, and sudden pain [2] that derives from the exposed cervical pulp–dentine complex. Ultimately, this pain may discourage the individual from consuming food and beverages, brushing their teeth, or even breathing [8].

Hypersensitive dentin can be treated with various procedures. Together with the elimination of nociceptive stimuli, two major treatment approaches stand out: (1) altering the nervous response by inhibiting or decreasing neuronal transmission and (2) occluding the permeable dentinal tubules [9]. Regarding potassium salts applications, potassium nitrate is the most preferred for use as a nervous modifier [10]. With regard to managing tubular occlusion, various methods are employed, including chemical interventions using fluorides [11], oxalates [12], or arginine [13,14] and physical interventions using adhesives [15] or laser therapies [16]. Low-power lasers (GaAlAs diode laser) have effects on neural transmission and medium-power lasers (Nd: YAG, CO and Er: YAG laser) reduce the diameter of dentinal tubules and cause occlusion. Because of this, these two approaches are preferred for eliminating dentin hypersensitivity [16,17]. Compared with conventional desensitizing topical agents, laser treatment methods lead to rapid results with less application time and less time spent for the patient [18]. However, laser treatments have certain disadvantages, such as their high cost and complexity of use [19]. Thus, recent studies have focused on identifying new materials and new treatment methods [20].

Nanohydroxyapatite resembles an inorganic bone structure, and it is biocompatible; therefore, it can be used for various dental purposes. Recently, interest in nanotechnology has produced promising applications in dentistry for nanohydroxyapatite, which offers crystals ranging in size from 50 to 1000 nm. The ability of nanohydroxyapatite to bind to proteins is due to the size of the nanoparticles, which noticeably extend the surface area to which proteins can bind. In addition, nanohydroxyapatite fills the small gaps and depressions on the enamel surface. It also has impressive remineralizing effects on initial enamel lesions and is more effective than fluorides in this regard [21]. Since it is a bioactive material and can enhance the mineralization process, this material has the potential to inhibit dentine hypersensitivity [22]. Several types of treatments for dentin hypersensitivity involve using products or laser applications directly on the affected dentin tissue. In this way, such treatments are likely to change the surface roughness of the dentin [23]. Bacterial adhesion and plaque retention on tooth structures might be accelerated due to an increase in surface roughness, and this can make the surface prone to caries and periodontal disease [24]. Existing research has limited data regarding the selection of reliable treatment modalities for dentin hypersensitivity [25]. Hence, this study aims to investigate the effects of a novel nanohydroxyapatite gel and Er:YAG laser on the surface roughness, surface morphology, and elemental content after dentin hypersensitivity treatments.

The null hypothesis of this study was as follows:

There will be no differences in surface roughness, dentinal tubule morphology, or elemental content after different dentin hypersensitivity treatments.

## 2. Materials and Methods

This in vitro study was approved by the local ethics committee (Bezmialem Vakif University Ethics Committee for Non-Invasive Studies, Process no: 2022/323). The specimen size was determined on the basis of the estimated effect size between groups, in accordance with the literature [26,27]. In this study, 10 specimens for surface roughness measurement and 5 specimens for elemental content analysis were necessary for each group to obtain a medium effect size (d = 0.50) using 95% power and a 5% type 1 error rate.

A total of 75 extracted sound human molars were used. Out of the 75 sound human molars, 50 were used to evaluate surface roughness and 25 out of the 75 sound human molars were used to evaluate surface morphology and elemental content. Teeth with caries, root resorption, cracks, fractures, or restorations were excluded. The teeth were cleaned with curettes and stored in distilled water until the experiment. Dentin specimens (2 × 3 × 3 mm^3^ area) were obtained using a model trimmer and high-speed diamond saw (MT3 Wet trimmer, Renfert GmbH, Hilzingen, Germany) under water irrigation. Enamel was cut up to 2 mm below the central fossa to expose the dentin parallel to the occlusal surface. A stereomicroscope (SMZ 1000, Nikon, Japan) was used to control the dentin specimens for lack of enamel. Additionally, the thickness of the specimen was verified with a digital caliper (Insize/China). The surfaces were polished using 400-, 600-, and 800-grit silicon carbide papers on a polishing device (Minitech 233, Presi, Grenoble, France) under water cooling (B.0.) [28].

Table 1 summarizes the materials used in this study and their compositions. The specimens were randomly divided into five groups according to different dentin hypersensitivity treatment methods:

**Group ID** (**Intact Dentin/positive control**): The specimen received no dentin exposure application and no dentin hypersensitivity treatment.

**Group DD** (**Demineralized Dentin/negative control):** The specimen received no dentin hypersensitivity treatment.

**Group BF** (**Bifluorid 10):** The fluoride varnish (Bifluorid 10, Voco GmbH, Cuxhoven, Germany) was applied to the specimen with a microbrush under dry conditions. It was allowed to be absorbed for 10–20 s and was then air-dried according to the manufacturers’ instructions.

**Group Lsr** (**Er:YAG laser):** The Er: YAG laser (Fotona AT Fidelis, Ljubljana, Slovenia) was applied to the specimen under the sets of parameters (50 mJ, 0.50 W, 10 Hz) with a wavelength of 2940 nm at 1 cm distance for 30 s [29]. A non-contact hand piece (H02) was applied perpendicular to the surfaces with a cylindrical sapphire optical fiber tip (1.3 mm in diameter, 8 mm in length). The tip was moved mesiodistally at a speed of approximately 1 mm/s.

**Group NHA** (**nanohydroxyapatite):** A novel nanohydroxyapatite-containing gel (Biodent Medical, Istanbul, Turkey) was applied to the specimen for 1 min at 500 rpm with a slow hand piece using a rubber cap under dry conditions according to the manufacturers’ instructions.

**Table 1:** The materials used in this study and their compositions.

Materials	Trade	Composition
**Bifluorid 10**	Voco GmBh, Cuxhoven, Germany	5% NaF, 5% CaF, (22.600 ppm)
**Nanohydroxiapatite containing gel**	Biodent Medical, Istanbul, Turkey	Nanohydroxyapatite particles with typical particle size below 50 nm in a rod-like shape (typically 30–40 nm length and 5–10 nm width).

To expose the dentinal tubules and simulate dentin hypersensitivity, 35% phosphoric acid (Scotchbond Universal Etchant, 3M ESPE, St. Paul, MN, USA) was applied to the surfaces for 1 min for all tested groups except Group ID. After that, etched specimens were rinsed with distilled water for 20 s. Then, all the etched and rinsed specimens were air-dried. All dentin hypersensitivity treatments were performed with a single operator for standardization (D.S.).

Next, 10 specimens from each group were assessed using a contact profilometer (Mahr GmbH, Marsurf PS1, Göttingen, Germany) for surface roughness (Ra, mm). The measurements were obtained from three different parts of the surface for each specimen with a stylus tip radius of 5 μm and a stylus driving speed of 0.5 mm/s, in accordance with EN ISO 4288.21. The arithmetic mean of these measurements was calculated. After measuring every five specimens, the tool was calibrated for reliable findings. A second operator (M.D.), who was blind to the dentin hypersensitivity treatment method, performed this surface roughness procedure [30,31].

Five specimens from each group were chosen, and the surface morphology and elemental content was evaluated via SEM (Thermo Fisher Scientific, Phenom XL, Waltham, MA, USA). These specimens were gold-sputter-coated to obtain the electrical conductivity necessary for SEM imaging. A CeB6 thermionic source using 5, 10, or 15 kV acceleration voltages provided illumination during SEM imaging, which achieved a resolution below 20 nm. The morphology was evaluated using a secondary electron detector, which monitored the geometrical differences on the surface in three dimensions. The micromorphology of the surfaces was completed at 5000× magnification. Then, the surface content was evaluated using the backscattered electron detector (BSD), which demonstrates chemical composition based on the density variances of different elemental contents.

For the chemical characterization of the dentin specimens, a semiquantitative chemical microanalysis method, namely energy-dispersive X-ray spectroscopy (EDS), was utilized. The EDS module of the scanning electron microscope (Phenom XL, Thermo Fisher Scientific) was selected, since this module has the ability to identify elements from boron (B) to americium (Am) by virtue of the ultrathin silicon nitride X-ray window. A thermoelectrically cooled (LN2-free) silicon drift detector was utilized, and an energy resolution below 137 eV at Mn Kα was achieved with a 10 eV/ch processing capability, with 2048 channels and 300,000 counts/second. Thus, an elemental analysis with high certainty was performed for the basic elements (C, O, F, P, Ca, Mg). A researcher who was blind to the dentin hypersensitivity treatment method performed all the SEM and EDS analyses.

### Statistical Analysis

Statistical analysis was performed using SPSS 23.0 for Windows (SPSS Inc., Chicago, IL, USA). The Shapiro–Wilk test was first used to indicate the normality of variables, and the data were then analyzed with Levene’s test for homogeneity of variances. The surface roughness data were normally distributed. Welch ANOVA was used to show differences between groups. Pairwise comparisons were performed via Games Howell test. Since EDS data did not satisfy parametric test assumptions, the data were analyzed with non-parametric tests. The Kruskal–Wallis test was performed to compare between-group differences in terms of EDS. Pairwise comparisons were developed via Dunn test. Statistical significance was determined at a confidence level of 0.05 in all analyses.

## 3. Results

### 3.1. Surface Roughness Evaluation

Mean surface roughness values and standard deviations are presented in Table 2. Group Lsr exhibited significantly lower surface roughness than group NHA and group BF (*p* ˂ 0.05). Moreover, no significant differences in surface roughness were found between the other tested groups (*p* ˃ 0.05).

### 3.2. Surface Morphology and Elemental Content Analysis

**Figure 1:**
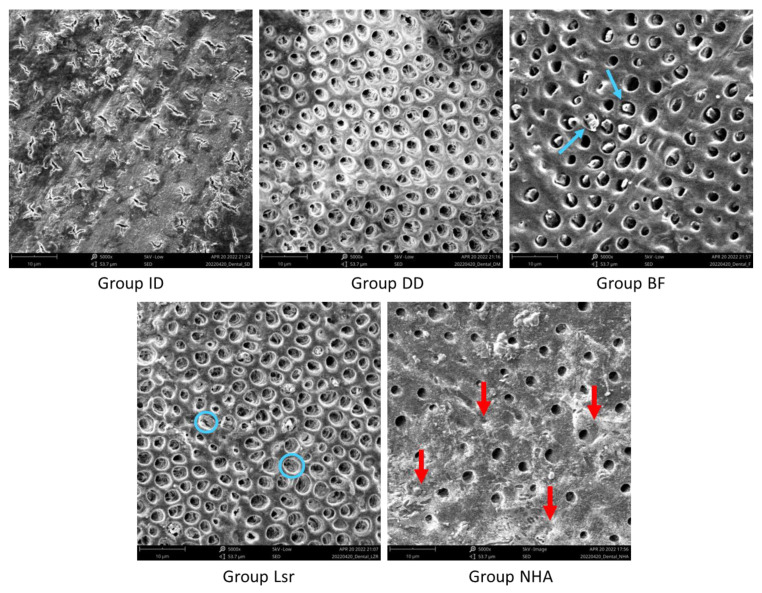
Representative SEM images of all tested groups. (Red arrows: deposit formation, blue arrows: precipant plugs, blue circles: occluded tubules with melting appearance).

Representative SEM images of all tested groups are given in Figure 1. The SEM analysis indicated that most of the dentinal tubules with deposit formations (red arrows) were obliterated for Group NHA. Additionally, precipitant plugs (blue arrows) with partially occluded dentinal tubules were observed in Group BF, while either partially or completely occluded tubules with a melting appearance (blue circle) were detected in Group Lsr.

**Figure 2:**
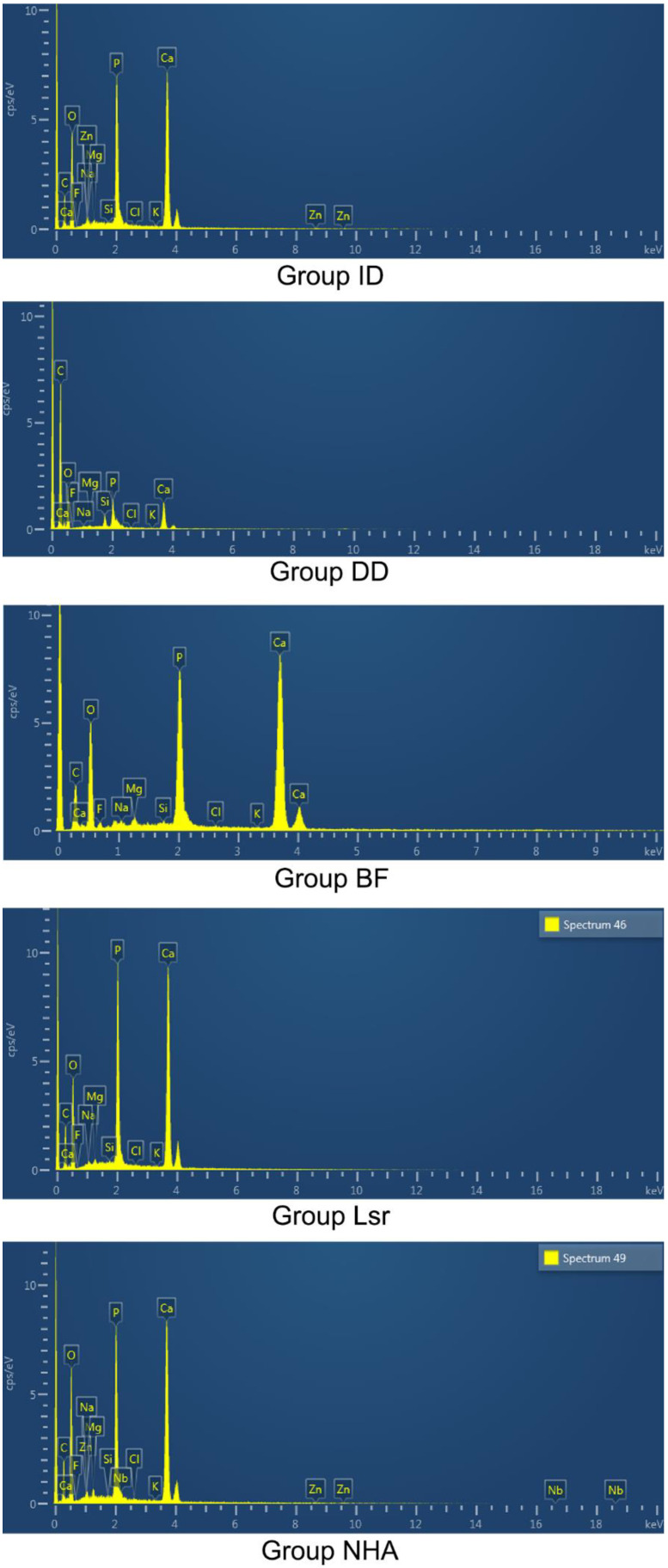
Representative semi-quantitative chemical analysis and distribution of elements of all tested groups.

The representative semiquantitative chemical analyses and distribution of elements for all tested groups are provided in Figure 2 and Table 3 (%). The EDS analysis revealed that phosphorus (P) and calcium (Ca) were the predominant elements. Significantly lower Ca and P amounts were found for Group DD compared with Group ID. Group NHA and Group Lsr yielded similar amounts of all tested elements to Group ID. Significant differences in the P and F amounts were detected among the treated groups. F amounts were found to be significantly higher in Group BF compared with Group Lsr, but similar to those in Group NHA. Significantly higher F amounts were found for Group BF compared with Group ID. The amount of Mg in Group NHA and Group Lsr was significantly higher than in Group DD. All treated groups showed a similar Mg amount to Group ID.

## 4. Discussion

In this study, the effects of a novel nanohydroxyapatite-containing gel and Er: YAG laser on surface roughness, surface morphology, and elemental content after dentin hypersensitivity treatments were evaluated. The null hypothesis that proposed that there would be no differences in surface roughness, dentinal tubule morphology, or elemental content after different dentin hypersensitivity treatments was rejected because the treated groups exhibited partial or complete occlusion of the dentinal tubules and because significant differences in P and F amounts were detected among treated groups. In addition, the laser-treated groups exhibited significantly lower surface roughness values compared with the other tested groups.

Rough surfaces favor plaque accumulation and maturation [32]. The influence of surface roughness on supragingival plaque supports the need for smooth surfaces with a low surface free energy to eliminate plaque accumulation, thereby decreasing the number of teeth with caries and periodontitis [33]. The contact profilometry method has advantages, since it is an easily accessible and cost-effective measurement of surface roughness [34].

Thus, in this study, surface roughness was analyzed with this method. The laser-treated group revealed the most favorable surface roughness values. The Er: YAG laser is one of the most preferred types of lasers to treat dental hard tissues because its wavelength (2940 nm) is in harmony with the main absorption peak of water, which is absorbed well in all biological tissues, including enamel and dentin. Absorption of water was 15 and 10,000 times higher with Er: YAG lasers than with CO_2_ and Nd: YAG lasers, respectively. Thus, as a result of the high water absorption peak in comparison with other commercially available lasers, Er: YAG lasers have often been preferred for treating dental and oral conditions [29]. Zhuang et al., who investigated the effects of Er: YAG lasers used at various power- and wavelength levels (0.5 W/50 mJ/10 Hz, 1 W/50 mJ/20 Hz, 2 W/100 mJ/20 Hz and 4 W/200 mJ/20 Hz) on dentinal tubule occlusion, intrapulpal heat, and pulpal tissue structure, reported that the parameters 0.5 W/50 mJ were suitable for proper tubule occlusion. Furthermore, they indicated that the accumulation of energy from laser treatments might damage pulp [35]. Thus, in this study, the Er: YAG laser parameters 0.5 W/50 mJ/10 Hz were used to treat dentin hypersensitivity.

This expansion process results in the ablation of the surrounding material [36]. Therefore, the surface roughness findings of laser-treated specimens could be attributable to the ablation of dentin tissue. In addition, differences in surface roughness were found to be insignificant between other tested groups.

In the present study, SEM and EDS analysis were chosen to examine the dentin tubule morphology and elemental content of the dentin surfaces. It has been well documented that the level of dentin hypersensitivity is dependent on the magnitude and potency of the dentinal tubules [5]. Furthermore, according to hydrodynamic theory, as the dentinal fluid movement diminishes, dentine hypersensitivity also decreases. Fluoride is used often in clinical settings and studies to decrease dentin hypersensitivity. The action mechanism of fluoride accounts for its efficiency, as fluoride is able to form precipitates and occlude the tubules [1]. It has been reported that bifluorid varnish containing sodium fluoride and calcium fluoride provided the occlusion of open tubules and precipitation of calcium fluoride or proteins [37]. This fact is consistent with the SEM images of the current experiment, which indicated that Bifluorid 10 caused precipitant plugs with partially occluded dentinal tubules. EDS analysis showed that the Biflorid 10-treated group had higher F levels than the laser-treated groups, but similar to those of the NHA-treated groups.

Roughly, 97% of tooth enamel and 70% of dentin consists of hydroxyapatite (HA) [38]. NHA can act as a template for mineral crystal nucleation and growth to form a dentin-like structure during the remineralization process. Calcium and phosphate ions from the oral environment may attach to the dentin surface during the precipitation and will fill the empty places in the crystal complex [39]. In this study, SEM analysis revealed that most of the tubules were occluded for the NHA-treated specimens. The sizes of NHA particles allowed the material to penetrate through microcracks to reach the dentinal tubules [40]. The increase in surface area associated with the reduction in NHA particle size may have resulted in increased penetration and deposition of these molecules into the dentinal tubules, resulting in large tubular occlusion [41]. The findings of this study are consistent with Vano et al. [42], who concluded that NHA-containing toothpaste was effective in treating dentin hypersensitivity. According to the SEM images of this study, the application of NHA was effective for the occlusion of the dentinal tubules. The application of an Er: YAG laser would be expected to reduce these fluid movements by evaporating the superficial parts of dentinal fluid [43]. These ablation and evaporation facilities of the laser could have enabled the obliteration and occlusion effects. Kurt et al. [44], who evaluated the efficacy of the Er: YAG laser and different dentin desensitizers on dentin permeability reduction, reported that the laser-treated groups performed best and that nanohydroxyapatite toothpaste can be considered an alternative therapeutic product. This finding is in line with the SEM images of the present study, which display partially or completely occluded tubules with a melting appearance for the laser-treated specimens.

The occlusion effect and crystalline deposits result from the presence of calcium phosphate in the form of hydroxyapatite. In this study, for the laser- and NHA-treated groups, the EDS analysis indicated that the dentin surface deposits had similar levels of calcium and phosphorous to intact dentin. These findings could have contributed to the promising tubule occlusion of these groups’ SEM images. Moreover, P amounts for these groups were significantly higher than the amount of Biflorid 10. This finding is in line with Contrera-Arriaga et al. [45], who reported similar P amounts after Er:YAG laser irradiation. Mg concentration is 1% (wt) in dentin tissue. Mg^2+^ is involved in the biomineralization of teeth and directly affects crystallization and pattern generation of the inorganic mineral phase [46]. EDS analysis highlighted that all treated groups showed similar Mg amounts to Group ID. The discovered Mg^2+^ could contribute to the positive effect on the remineralization potentials of those desensitizing agents.

Regarding the limitations of the current in vitro study, only the early-term effects of BF, Lsr, and NHA were evaluated via SEM EDS and contact profilometry analysis, and the long-term effects were not examined. In addition, the erosion–abrasion cycle step was not used in our study. Further damage and different occlusion rates on dentinal tubules could be expected with such an approach. What is more, future studies could focus on combination therapies for dentinal tubule occlusion. Different specimen groups treated with dentin hypersensitivity agents such as NHA and subsequent Lsr application and other combination approaches might provide promising results.

## 5. Conclusions

Within the limitations of this study, it can be concluded that all dentin hypersensitivity treatment methods could provide promising results in terms of tubular occlusion efficiency. However, laser treatment resulted in smoother surfaces, which could help to prevent dental plaque accumulation.

## Figures and Tables

**Table 2:** Mean surface roughness values and standard deviations (±) of all tested groups (Ra, μm).

Group	Surface Roughness (±)	*p*
**Group ID**	0.865 ± 0.289 ^ab^	0.015
**Group DD**	1.121 ± 0.355 ^ab^	0.015
**Group BF**	1.262 ± 0.419 ^a^	0.015
**Grup Lsr**	0.856 ± 0.094 ^b^	0.015
**Group NHA**	1.155 ± 0.319 ^a^	0.015

The lowercase letters indicate significant differences between groups.

**Table 3:** The chemical distribution of elements for all tested groups (%).

	O	P	Ca	F	C	Mg
**Group ID**	52.59 ± 1 ^b^	12.06 ± 0.81 ^b^	23.43 ± 1.3 ^b^	0.04 ± 0.07 ^a^	10.16 ± 0.95 ^b^	0.26 ± 0.1 ^ab^
**Group DD**	68.46 ± 0.72 ^a^	2.41 ± 0.52 ^ac^	4.83 ± 0.94 ^a^	0.02 ± 0.04 ^a^	23.6 ± 0.61 ^a^	0.06 ± 0.02 ^a^
**Group BF**	55.41 ± 2.53 ^ab^	1.16 ± 0.17 ^a^	19.02 ± 3.15 ^ab^	1.07 ± 0.36 ^b^	13.2 ± 2.01 ^ab^	0.43 ± 0.04 ^b^
**Group Lsr**	57.28 ± 2.41 ^ab^	9.25 ± 1.43 ^bc^	18.74 ± 3.02 ^ab^	0.03 ± 0.08 ^a^	14.1 ± 2.03 ^ab^	0.23 ± 0.03 ^ab^
**Group NHA**	55.95 ± 2.86 ^ab^	9.76 ± 1.51 ^bc^	19.39 ± 3.48 ^ab^	0.26 ± 0.17 ^ab^	13.13 ± 2.34 ^ab^	0.4 ± 0.05 ^b^
***p***	<0.001	<0.001	<0.001	<0.001	<0.001	<0.001

The lowercase letters indicate significant differences between groups.

## Data Availability

Not applicable.
